# Survival after wake-up stroke and unknown-onset stroke—a nationwide observational study from the Norwegian Stroke Registry

**DOI:** 10.1093/esj/aakaf016

**Published:** 2026-01-01

**Authors:** Mary-Helen Søyland, Arnstein Tveiten, Agnethe Eltoft, Halvor Øygarden, Torunn Varmdal, Bent Indredavik, Ellisiv B Mathiesen

**Affiliations:** Department of Neurology, Hospital of Southern Norway, Kristiansand, Norway; Department of Clinical Medicine, UiT The Arctic University of Norway, Tromsø, Norway; Department of Neurology, Hospital of Southern Norway, Kristiansand, Norway; Department of Clinical Medicine, UiT The Arctic University of Norway, Tromsø, Norway; Department of Neurology, University Hospital of North Norway, Tromsø, Norway; Department of Neurology, Hospital of Southern Norway, Kristiansand, Norway; Institute of Clinical Medicine, University of Oslo, Oslo, Norway; Department of Circulation and Medical Imaging, Norwegian University of Science and Technology, Trondheim, Norway; Department of Medical Quality Registries, St. Olav’s Hospital, Trondheim University Hospital, Trondheim, Norway; Department of Medical Quality Registries, St. Olav’s Hospital, Trondheim University Hospital, Trondheim, Norway; Department of Medicine, St. Olav’s Hospital, Trondheim University Hospital, Trondheim, Norway; Department of Neuromedicine and Movement Science, Norwegian University of Science and Technology, Trondheim, Norway; Department of Clinical Medicine, UiT The Arctic University of Norway, Tromsø, Norway; Department of Neurology, University Hospital of North Norway, Tromsø, Norway

**Keywords:** cerebrovascular disease/stroke, ischaemic stroke, wake-up stroke, unknown-onset stroke, survival, mortality, epidemiology, registry

## Abstract

**Introduction:**

The risk of death increases significantly after stroke as shown in previous studies from the general stroke population; however, specific knowledge regarding survival after wake-up stroke and unknown-onset stroke is lacking. We aimed to report short- and long-term survival after ischaemic stroke, by mode of onset, using data from a high-quality nationwide stroke registry.

**Patients and methods:**

Data from the Norwegian Stroke Registry for the period 2014-2023 were retrieved to assess short- and long-term survival after first-ever ischaemic stroke. Short-term survival was defined as surviving the first 30 days after stroke, and long-term survival was examined in 30-day survivors. Kaplan–Meier survival probabilities were estimated at 30 days, 1, 3 and 5 years after stroke for all patients and stratified by mode of onset: known-onset stroke, wake-up stroke and unknown-onset stroke. The relationship between mode of onset and all-cause mortality was assessed using multivariable regression models.

**Results:**

Of the 68,025 patients included, 45,084 had known-onset stroke, 12,429 wake-up stroke and 10,512 unknown-onset stroke. The 30-day survival rate was 91.3% for known-onset stroke, 92.4% for wake-up stroke and 91.7% for unknown-onset stroke, while 5-year survival rate among 30-day survivors was 65.4%, 67.6% and 60.4%, respectively. For 30-day survivors, using known-onset stroke as reference group, the adjusted HR for all-cause mortality in the total observation period for wake-up stroke was 0.99 (95% CI, 0.95–1.04), *P* = .778 and for unknown-onset stroke the HR was 1.19 (95% CI, 1.14–1.25), *P* < .001.

**Conclusion:**

Short-term survival was similar across all modes of onset, while unknown-onset stroke was associated with poorer long-term survival.

## Graphical Abstract

**Graphical Abstract:**
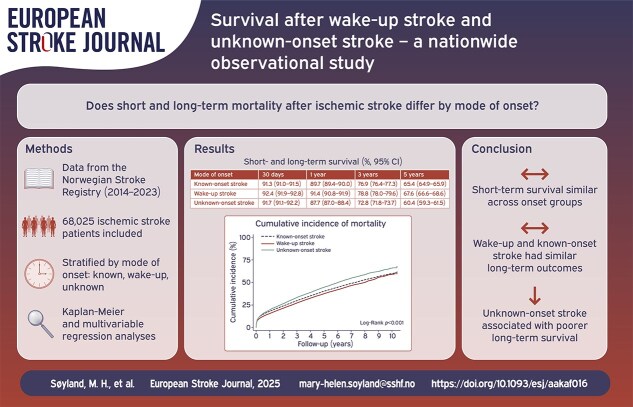

## Introduction

Stroke is a major contributor to loss of life expectancy, and although stroke-related mortality has declined considerably over the recent decades, it remains the second most common cause of death globally.^1–3^ Each year in Norway close to 9000 people are admitted to hospital with an acute stroke.^4^ One in 5 of these are wake-up strokes and 1 in 6 unknown-onset strokes.^5^ The current knowledge on survival after stroke is from the general stroke population including both acute ischaemic stroke (AIS) and ICH, or subgroups such as first-ever stroke or ischaemic stroke. To our knowledge, there are no previous publications reporting survival after ischaemic wake-up stroke, and no information on potential differences in survival between stroke subgroups by mode of onset such as known-onset stroke, wake-up stroke and unknown-onset stroke. Survival after ischaemic stroke is known to vary depending on stroke severity and access to acute treatment and care.^6–8^ These factors, along with the presence of immediate complications, are key determinants of short-term survival. In contrast, long-term survival reflects the broader effects of stroke, including the impact of comorbidities, and provides insights into the lasting effectiveness of early interventions. To what extent mode of onset affects survival is yet to be determined.

The aim of this research was to evaluate overall survival after first-ever ischaemic stroke and examine disparities in short- and long-term survival between known-onset stroke, wake-up stroke and unknown-onset stroke.

**Figure 1:**
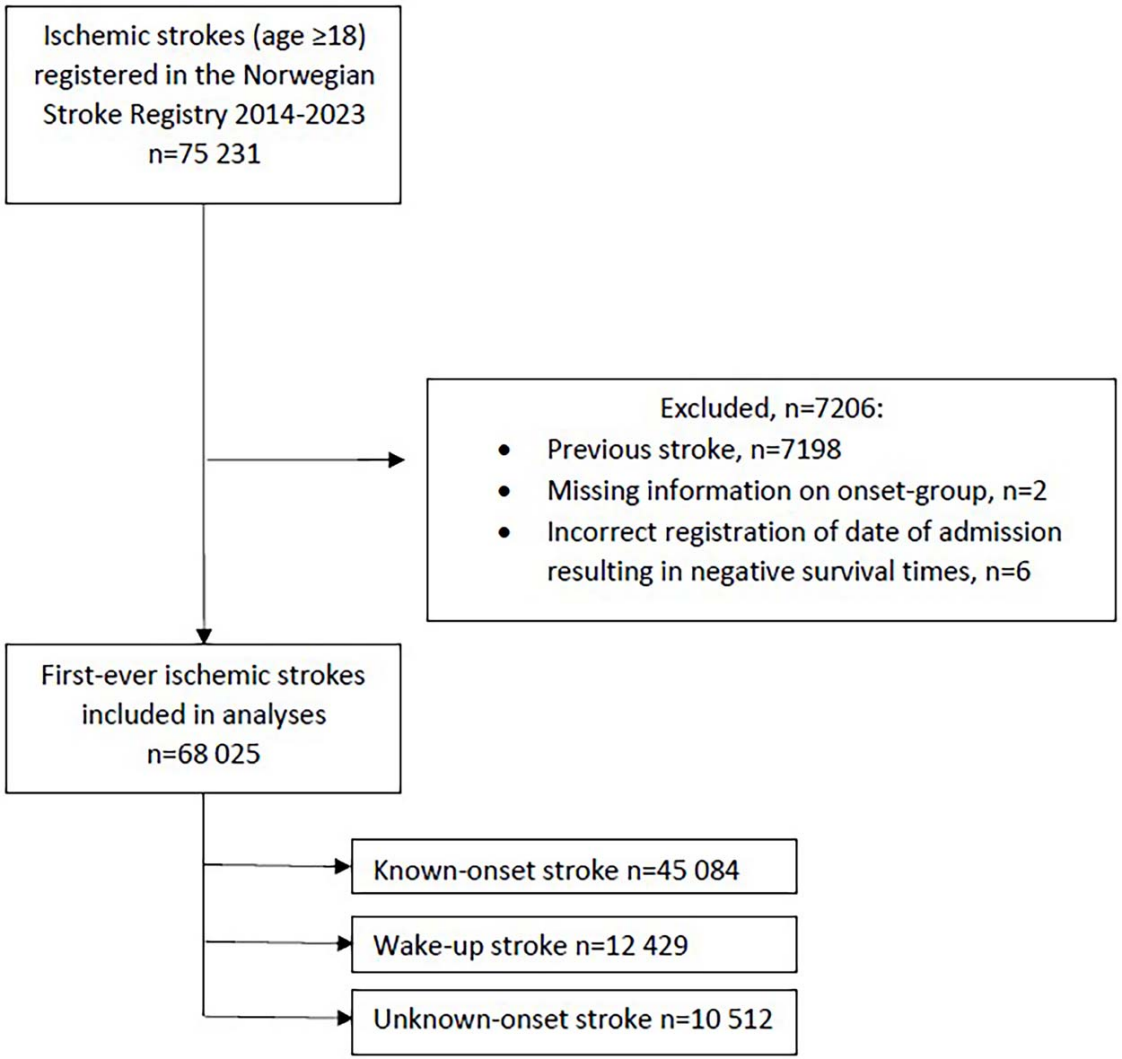
Patient selection.

## Patients and methods

This study used data from the Norwegian Stroke Registry from 2014 through 2023. The Norwegian Stroke Registry is a mandatory nationwide medical quality registry including all patients aged 18 years and older hospitalised for acute stroke in Norway. The registry has high coverage (87%), and has been validated as a reliable source of data for research.^9^ The International Classification of Diseases (ICD)-10 stroke diagnosis code is assigned by a treating physician, and data are manually registered via web-based forms by trained healthcare workers. Time of death is obtained from the Norwegian National Population Register and integrated into the Norwegian Stroke Registry continuously. Our data include death dates up until 31st May 2024. Information on cause of death is not available in the registry.

Patients included in this study were those with first-ever ischaemic stroke (ICD-10 code I63) (*n* = 68,033). Information on mode of onset was lacking in 1 patient, who was excluded. Six patients had survival times below zero suggesting incorrect registration in the registry. These were also excluded (Figure 1).

Information obtained from the Norwegian Stroke Registry included age, sex, stroke severity, atrial fibrillation, diabetes, previous myocardial infarction, use of antihypertensive medication and smoking. The use of antihypertensive medication was employed as proxy for hypertension. We also extracted information on discharge medications including the use of antiplatelet agents, anticoagulation, antihypertensives and lipid-lowering drugs. Stroke severity was assessed using the NIHSS. Information on ischaemic stroke subtype (eg, TOAST Classification) is not available in the registry.

Study approval was granted by both the Regional Committee for Medical and Health Research Ethics (REC) and the data protection officials at the Hospital of Southern Norway. Individual patient consent was not required due to the mandatory nature of the registry. The reporting of this study complies with the Strengthening the Reporting of Observational studies in Epidemiology (STROBE) statement.

Data from the Norwegian Stroke Registry are accessible through Helsedata at https://helsedata.no, upon application.

### Outcome measures

We assessed short- and long-term survival after index ischaemic stroke by mode of onset. Short-term survival was defined as surviving the first 30 days after stroke. Long-term survival was examined in 30-day survivors at 1-, 3- and 5-years post-stroke, as well as across the entire follow-up period, which extends up to 10 years for some patients.

### Statistical analyses

Patient characteristics are presented for all patients and according to mode of onset groups. Results are presented as average and SD for age, median and IQR for NIHSS on admission and percentages for the remaining variables.

Survival time was defined as time from hospital admission to 31st May 2024, or, to death by any cause, whichever came first.

For comparison of survival between mode of onset groups, the Kaplan–Meier method was used to create cumulative mortality curves, as well as generate lifetables from the survival estimates. The log-rank test was used to examine between-group differences. Additional Kaplan–Meier survival analysis was performed with intravenous thrombolytic treatment (IVT) as a stratification factor.

Binary logistic regression was used to assess the association between mode of onset and surviving the first 30 days. For long-term survival in 30-day survivors, Cox proportional hazard regression was used. Known-onset stroke was defined as the reference group in both analyses. Results are presented as odds ratios (ORs) and hazard ratios (HRs) for all-cause mortality, respectively, with corresponding 95% confidence intervals (CIs), adjusted for age, sex, stroke severity (NIHSS), atrial fibrillation, diabetes, previous myocardial infarction, use of antihypertensive medication and smoking. The model was set using existing knowledge, not computerised calculations, and did not include any interaction terms. For the variable smoking, non-smokers (never) were selected as reference group, and for sex, males were defined as the reference group. Based on univariate analyses and Kaplan–Meier curves, we concluded that the proportional hazard assumption was sufficiently met for all variables included in the Cox proportional hazard regression.

We had < 1% missing for most variables, except for smoking (15.9%) and NIHSS on admission (18.7%) (Supplementary Materials S1 and S2). Missing data for the variable NIHSS on admission differed substantially by mode of onset: 16% in both the known-onset and wake-up stroke groups, compared to 34% in the unknown-onset stroke group (Supplementary Material S3).

Furthermore, we performed sensitivity analyses including and excluding NIHSS on admission in the regression analyses, due to the high number of missing values for this variable (Supplementary Material S4). We also conducted interaction analyses for onset and sex, as well as separate regression analyses for men and women to assess potential sex-related differences in survival outcomes.

Stata/SE (v.17.0) was used for statistical analyses.

## Results

We included 68,025 patients with first-ever ischaemic stroke registered in the Norwegian Stroke Registry from 2014 to 2023. Of these, 45,084 had known-onset stroke, 12,429 wake-up stroke and 10,512 unknown-onset stroke. The baseline characteristics of included patients by mode of onset group are listed in Table 1.

**Figure 2:**
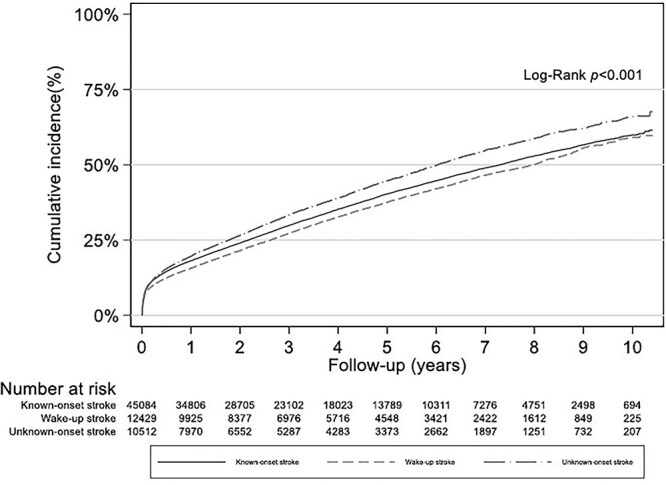
Cumulative incidence of death, stratified by mode of stroke onset.

**Table 1:** Characteristics of included patients

	**All patients (*n* = 68,025)**	**Known-onset stroke (*n* = 45,084)**	**Wake-up stroke (*n* = 12,429)**	**Unknown-onset stroke (*n* = 10,512)**
Age, mean (SD)	73.8 (13.2)	73.7 (13.3)	73.4 (13.1)	74.5 (12.8)
Female	44.9	45.0	43.0	46.7
Living arrangement				
Lives with someone	56.8	57.9	60.2	48.3
Lives alone	39.9	38.8	37.1	48.1
Institution	3.2	3.3	2.8	3.6
mRS prior to stroke^a^				
0–1	76.5	76.6	78.9	73.1
2–5	23.5	23.4	21.1	26.7
Smoking^a^				
No	44.7	45.3	42.8	44.5
Current	23.6	22.6	25.1	26.0
Previous	31.8	32.2	32.1	29.5
Diabetes	18.8	18.3	18.2	21.7
AF	24.7	25.2	23.3	24.3
Previous MI	13.4	13.5	13.5	12.9
Use of anti-hypertensives	54.5	54.4	54.2	55.3
NIHSS on admission^a^, median (IQR)	3 (1–7)	3 (1–7)	3 (1–6)	2 (1–5)
IVT	20.4	27.7	10.4	1.0

^a^> 5% missing values.

Age is presented as mean (SD), NIHSS as median (IQR), all other results as percentages.

Abbreviations: IVT = intravenous thrombolytic treatment; MI = myocardial infarction.

The cumulative incidence of death among patients with known-onset stroke, wake-up stroke and unknown-onset stroke is shown in Figure 2. Unadjusted log-rank tests revealed statistically significant differences in cumulative mortality distributions across all 3 groups (*P* < .001), as well as in pairwise comparisons between known-onset and wake-up strokes (*P* < .001) and between known-onset and unknown-onset strokes (*P* < .001) (Supplementary Material S5). Survival at 30 days was similar in all mode of onset groups, ranging from 91.3% in the known-onset group to 91.7% in the unknown-onset group and 92.4% in the wake-up stroke group (Table 2). This translates to 30-day case fatality rates of 8.7% for known-onset stroke ([3931/45,084]; 95% CI, 0.08–0.09), 7.6% for wake-up stroke ([946/12,429]; 95% CI, 0.07–0.08) and 8.4% for unknown-onset stroke ([878/10,512]; 95% CI, 0.08–0.09). There was no difference in mortality between groups after adjustment for age, sex, cardiovascular risk factors and stroke severity (Table 3).

**Table 2:** Proportion of survival after 30 days and 1, 3 and 5 years in patients with ischaemic known-onset, wake-up and unknown-onset stroke

	***n***	**30 days**	**1 year**	**3 years**	**5 years**
Overall	68,025	91.5 (91.3–91.8)	89.7 (89.5–90.0)	76.6 (76.2–76.9)	65.0 (64.6–65.5)
Mode of onset					
Known-onset stroke	45,084	91.3 (91.0–91.5)	89.7 (89.4–90.0)	76.9 (76.4–77.3)	65.4 (64.9–65.9)
Wake-up stroke	12,429	92.4 (91.9–92.8)	91.4 (90.8–91.9)	78.8 (78.0–79.6)	67.6 (66.6–68.6)
Unknown-onset stroke	10,512	91.7 (91.1–92.2)	87.7 (87.0–88.4)	72.8 (71.8–73.7)	60.4 (59.3–61.5)

*n*; number of stroke cases.

Survival at 30 days, and for 30-day survivors at 1, 3 and 5 years, are presented as percentages % with (95% CI). Proportions are derived from the Kaplan–Meier survivor estimates.

**Table 3:** Multivariable analyses of risk of short- and long-term mortality in patients with ischaemic stroke

	**Short-term mortality *n* = 46,456**			**Long-term mortality *n* = 43,983** ^**a**^		
	**OR**	**95% CI**	***P* value**	**HR**	**95% CI**	***P*-value**
Onset						
Known-onset stroke (reference)	–	–	–	–	–	–
Wake-up stroke	0.97	0.86–1.09	.597	0.99	0.95–1.04	.778
Unknown-onset stroke	1.02	0.88–1.18	.783	1.19	1.14–1.25	<.001
Age	1.08	1.07–1.08	<.001	1.10	1.09–1.10	<.001
Female	0.99	0.90–1.10	.904	0.92	0.88–0.95	<.001
Atrial fibrillation	1.12	1.02–1.24	.020	1.27	1.22–1.31	<.001
Previous MI	1.37	1.22–1.54	<.001	1.26	1.21–1.32	<.001
Diabetes	1.09	0.97–1.23	.160	1.35	1.30–1.41	<.001
Antihypertensive medication	0.98	0.89–1.09	.760	1.06	1.03–1.10	.001
Smoking						
Never (reference)	–	–	–	–	–	–
Previous	1.01	0.91–1.12	.885	1.18	1.14–1.23	<.001
Current	1.12	0.98–1.29	.107	1.67	1.59–1.75	<.001
NIHSS on admission	1.18	1.17–1.18	<.001	1.05	1.05–1.06	<.001

^a^Patients who died within 30 days after onset were excluded from analysis of long-term survival. First 30 days were analysed using logistic regression, while Cox regression was used for analyses of 30-day survivors.

Abbreviations: HR = hazard ratio; MI = myocardial infarction; OR = odds ratio.

Among 30-day survivors, patients with wake-up stroke had the numerically highest survival rates after 1, 3 and 5 years (91.4%, 78.8%, 67.6%, respectively), closely followed by known-onset stroke (89.7%, 76.9%, 65.4%). Unknown-onset stroke had the lowest survival rates overall (87.7%, 72.8%, 60.4%) (Table 2). The poorer long-term survival outcomes observed for the unknown-onset stroke group remained statistically significant in the adjusted Cox regression analyses with a HR for all-cause mortality of 1.19 (95% CI, 1.14–1.25), *P* < .001 (Table 3). The adjusted HR for all-cause mortality in wake-up stroke was 0.99 (95% CI, 0.95–1.04), *P* = .778 (Table 3). Treatment with antiplatelets, anticoagulants, antihypertensives and lipid-lowering drugs at discharge was similar in the 3 groups (Supplementary Material S6), and further adjustment for these variables did not change the results.

**Figure 3:**
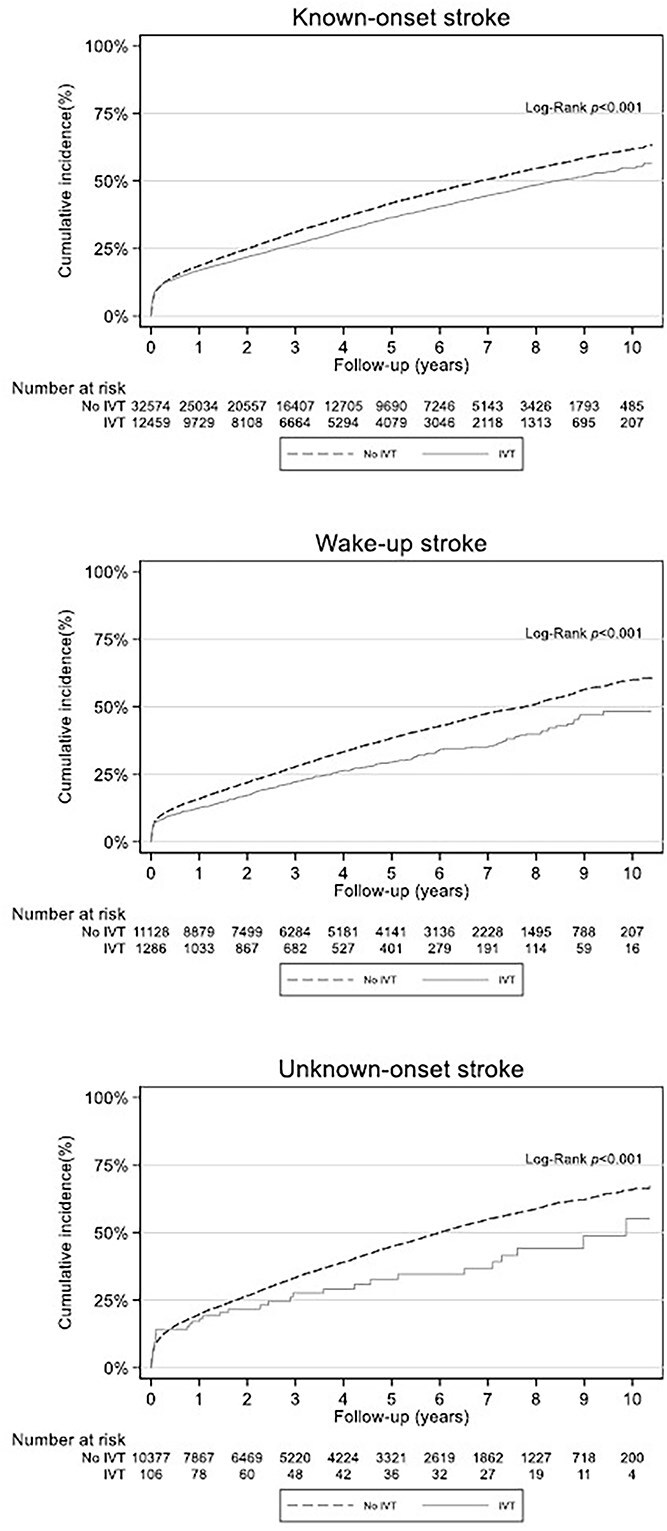
Cumulative incidence of death within stroke onset groups, stratified by intravenous thrombolytic treatment (IVT). The median (IQR) onset-to-treatment time in patients with known onset stroke was 112 (79–164) minutes.

Kaplan–Meier curves for the individual mode of onset groups comparing patients who received IVT with those not treated with IVT showed lower cumulative mortality in the treatment groups (Figure 3). This finding was consistent across all modes of onset.

In sex-specific multivariable adjusted analyses unknown-onset stroke had a poorer long-term survival for both men and women, while short-term survival was similar across all modes of onset, for both sexes. Women had a more favourable long-term survival compared to men with a HR for all-cause mortality of 0.92 (95% CI, 0.88–0.95) *P* < .001, while there was no difference between sexes for short-term survival with an OR for women of 0.99 (95% CI, 0.90–1.10), *P* = .904 (Supplementary Materials S7–S9). When testing for interaction between onset and sex, a statistically significant interaction effect was found for short-term survival in wake-up stroke, *P* = .045.

In sensitivity analyses, when NIHSS on admission was excluded from the multivariable regression model, wake-up stroke had a favourable short- and long-term survival, while the poorer long-term survival seen in unknown onset stroke remained the same (Supplementary Material S4). Baseline characteristics for patients with missing information on the variable NIHSS on admission were similar to those seen for all included patients. Patients with missing information on smoking status, however, were older, more often female, lived alone or in an institution, had a higher pre-stroke mRS and NIHSS on admission and a higher proportion of atrial fibrillation (Supplementary Material S10). Excluding smoking from the multivariable regression model did not alter the survival outcomes.

## Discussion

This is the first nationwide registry-based study on survival after ischaemic wake-up stroke and unknown-onset stroke. Our main finding was that unknown-onset stroke was associated with poorer long-term survival when compared to known-onset stroke and wake-up stroke, while the latter 2 had similar survival outcomes.

Updated results on long-term survival in ischaemic stroke patients from comparable recent surveys are scarce. Previous studies with observation periods between the mid-1980s and 2010 showed 5-year survival rates ranging from 40% to 64% in all strokes.^10–13^ In a previous Lithuanian study of ischaemic stroke patients aged 25–64 years who were included between 1986 and 2011, 77% survived 5 years.^14^ The 5-year survival in a Swedish nationwide registry-based study from 2011 to 2013 was 49%.^15^ Considering the reduction in death rates from stroke in the past decade,^2^ it is likely that the earlier observation period can explain the lower survival rate in the Swedish study. The somewhat higher mean age compared to our study (76.4 vs 73.8 years) may also have contributed to the differences.

We found no significant difference between known-onset stroke and wake-up stroke in neither unadjusted nor adjusted analyses. When comparing cumulative mortality curves by mode of onset, the proximity of the curves for wake-up stroke and known-onset stroke indicate that survival is similar in these 2 groups. This finding aligns with previous studies revealing similar characteristics and functional outcomes of wake-up stroke and known-onset stroke. In light of these prior findings, the comparable survival observed here is not unexpected and reinforces the similarity between these 2 groups.^5^^,^^16–19^ Our findings, however, indicate a significant disparity in long-term survival outcomes depending on the timing of stroke onset, highlighting a less favourable prognosis for patients with unknown-onset stroke. This may be related to these patients having a poorer functional status prior to the stroke, more often living alone, and to some extent having an increased burden of comorbidity.

There was a trend towards a better survival outcome for patients treated with IVT across all mode of onset groups as illustrated by the cumulative mortality curves in Figure 3. Our study was not designed to assess treatment effect, but illustrates a tendency towards a positive effect of treatment. This finding, from our population-based study, is in line with the survival benefit presented previously by Berge et al. from the IST-3 trial data, showing increased long-term survival after IVT.^6^ On the other hand, Emberson et al. found no difference in 90-day mortality between thrombolysed patients vs controls.^20^

There are several strengths in our study. Firstly, the large sample size and high statistical power enhance the robustness of our findings. Also, the high-quality registry with near complete coverage ensures comprehensive data capture. The long follow-up period allows for assessment of both short- and long-term mortality rates. Death as a main outcome is definitive and unambiguous with the Norwegian National Population Register providing complete and reliable outcome information. Lastly, the use of well-established statistical methodologies strengthens the quality of our results, providing a solid foundation for the conclusions drawn.

Limitations in our study include missing data for the variables smoking and NIHSS on admission, which may introduce some degree of bias. Furthermore, the unknown-onset stroke group possibly comprises a heterogeneous population where potential reasons for unknown onset status may include patients being unable to accurately recall the exact time of symptom onset, either due to pre-stroke cognitive comorbidity or the current stroke symptoms, alternatively the information may not be available due to inadequate medical record-keeping. This is further illustrated by the higher proportion of missing NIHSS information for this group compared to the other 2 groups. Another limitation is the high proportion of mild strokes in the Norwegian population potentially limiting the generalisability of our findings for some countries. Lastly, as with all registry-based studies there is the possibility of variations in reporting accuracy on baseline characteristics across hospitals.

## Conclusion

In this large nationwide registry-based study, short-term survival was similar across mode of onset groups. In multivariable adjusted analysis, long-term survival was significantly lower in unknown-onset stroke patients compared to known-onset stroke, while no difference was found between patients with known-onset stroke and wake-up stroke.

## Supplementary Material

aakaf016_STROBE_checklist

aakaf016_Supplemental_material_revised_clean

## Data Availability

Data from the Norwegian Stroke Registry are accessible through Helsedata at https://helsedata.no, upon request.
