# Supplementary material for: Survival after wake-up stroke and unknown-onset stroke—a nationwide observational study from the Norwegian Stroke Registry
Source: Eur Stroke J. 2026 Jan 1;11(1):aakaf016. doi: 10.1093/esj/aakaf016 (PMC12866631; doi:10.1093/esj/aakaf016)
Supplement: aakaf016_Supplemental_material_revised_clean [file aakaf016_supplemental_material_revised_clean.docx]

## Supplemental materials

## S1. Missing data

| Variable, n (%) | Included patients, n=68 025 |
| --- | --- |
| Age | 0 |
| Sex | 0 |
| Mode of onset | 0 |
| Prior myocardial infarction | 668 (1.0) |
| Diabetes | 414 (0.6) |
| Atrial fibrillation | 604 (0.9) |
| Antihypertensive drugs | 656 (1.0) |
| Smoking | 10 815 (15.9) |
| mRS prior to stroke | 4521 (6.7) |
| Living arrangement prior to stroke | 1902 (2.8) |
| NIHSS on admission | 12 702 (18.7) |
| Thrombolytic treatment | 95 (0.1) |

mRS: modified Rankin Scale, NIHSS: National Institute of Stroke Scale.

## S2. Percentage of missing data by survival status

|  | All included patients  n=68 025 | Survivors  n=39 771 | Died  n=28 254 | Died ≤ 30days  n=5755 |
| --- | --- | --- | --- | --- |
| Age | 0 | 0 | 0 | 0 |
| Sex | 0 | 0 | 0 | 0 |
| Mode of onset | 0 | 0 | 0 | 0 |
| Prior myocardial infarction | 1.0 | 0.8 | 1.3 | 2.1 |
| Diabetes | 0.6 | 0.6 | 0.7 | 1.3 |
| Atrial fibrillation | 0.9 | 0.8 | 1.1 | 1.9 |
| Antihypertensive drugs | 1.0 | 0.8 | 1.2 | 2.2 |
| Smoking | 15.9 | 11.1 | 22.6 | 37.7 |
| mRS prior to stroke | 6.7 | 5.2 | 8.6 | 12.6 |
| NIHSS on admission | 18.7 | 15.3 | 23.4 | 30.2 |
| Thrombolytic treatment | 0.1 | 0.1 | 0.2 | 0.1 |

mRS: modified Rankin Scale, NIHSS: National Institute of Stroke Scale.

## S3. Percentage of missing data by mode of onset

|  | All included patients  n=68 025 | KOS  n=45 084 | WUS  n=12 429 | UOS  n=10 512 |
| --- | --- | --- | --- | --- |
| Age | 0 | 0 | 0 | 0 |
| Sex | 0 | 0 | 0 | 0 |
| Prior myocardial infarction | 1.0 | 1.0 | 0.8 | 1.2 |
| Diabetes | 0.6 | 0.6 | 0.5 | 0.7 |
| Atrial fibrillation | 0.9 | 0.9 | 0.8 | 0.9 |
| Antihypertensive drugs | 1.0 | 1.0 | 0.8 | 1.2 |
| Smoking | 15.9 | 16.3 | 13.7 | 16.7 |
| NIHSS on admission | 18.7 | 15.9 | 15.7 | 33.9 |

KOS: known-onset stroke, WUS: wake-up stroke, UOS: unknown-onset stroke, NIHSS: National Institute of Stroke Scale.

## S4. Multivariable analyses of risk of short- and long-term mortality in patients with ischemic stroke without adjustment for NIHSS.

|  | Short-term survival  n= 56 131 | | | Long-term survival  n= 52 684* | | |
| --- | --- | --- | --- | --- | --- | --- |
|  | OR | 95% CI | P value | HR | 95% CI | P value |
| Onset |  |  |  |  |  |  |
| Known-onset stroke (reference) | - | - |  | - | - |  |
| Wake-up stroke | 0.87 | 0.79-0.96 | 0.004 | 0.95 | 0.91-0.99 | 0.008 |
| Unknown-onset stroke | 0.92 | 0.83-1.01 | 0.084 | 1.15 | 1.11-1.20 | <0.001 |
| Age | 1.07 | 1.07-1.08 | <0.001 | 1.09 | 1.09-1.10 | <0.001 |
| Female | 1.21 | 1.12-1.30 | <0.001 | 0.94 | 0.91-0.97 | <0.001 |
| Atrial fibrillation | 1.70 | 1.57-1.83 | <0.001 | 1.37 | 1.33-1.42 | <0.001 |
| Previous MI | 1.53 | 1.40-1.67 | <0.001 | 1.27 | 1.22-1.32 | <0.001 |
| Diabetes | 1.08 | 0.99-1.18 | 0.099 | 1.35 | 1.31-1.41 | <0.001 |
| Antihypertensive medication | 0.98 | 0.85-0.99 | 0.034 | 1.04 | 1.01-1.08 | 0.007 |
| Smoking |  |  |  |  |  |  |
| Never (reference) | - | - | - | - | - | - |
| Previous | 1.02 | 0.94-1.11 | 0.610 | 1.17 | 1.13-1.22 | <0.001 |
| Current | 1.24 | 1.11-1.38 | <0.001 | 1.67 | 1.60-1.74 | <0.001 |

Patients who died within 30 days after onset were excluded from analysis of long-term survival. First 30 days were analyzed using logistic regression, while Cox regression was used for analyses of 30-day survivors.

OR: odds ratio, HR: hazards ratio, CI: confidence interval

## S5. Pairwise comparison of cumulative incidence of death

## S6. Discharge medications by stroke onset group

Results presented as percentages.

|  | All patients | Known-onset stroke | Wake-up stroke | Unknown-onset stroke |
| --- | --- | --- | --- | --- |
| Anti-platelet therapy | 70.6 | 70.2 | 72.6 | 70.2 |
| Oral anticoagulation | 24.9 | 25.5 | 23.7 | 23.6 |
| Statin | 74.3 | 74.4 | 76.5 | 70.9 |
| Anti-hypertensives | 60.4 | 60.3 | 60.4 | 61.0 |

## S7. Cumulative incidence of death after first-ever ischemic stroke in men and women

## S8. Multivariable adjusted analysis of risk of short- and long-term mortality after ischemic stroke in men

|  | Short-term survival  n= 26 284 | | | Long-term survival  n= 25 172* | | |
| --- | --- | --- | --- | --- | --- | --- |
|  | OR | 95% CI | P value | HR | 95% CI | P value |
| Onset |  |  |  |  |  |  |
| Known-onset stroke (reference) | - | - | - | - | - | - |
| Wake-up stroke | 0.85 | 0.71-1.02 | 0.076 | 0.97 | 0.91-1.02 | 0.240 |
| Unknown-onset stroke | 1.17 | 0.95-1.45 | 0.134 | 1.20 | 1.12-1.29 | <0.001 |
| Age | 1.07 | 1.07-1.08 | <0.001 | 1.10 | 1.09-1.10 | <0.001 |
| Atrial fibrillation | 1.07 | 0.92-1.23 | 0.387 | 1.25 | 1.19-1.32 | <0.001 |
| Previous MI | 1.39 | 1.19-1.63 | <0.001 | 1.24 | 1.17-1.31 | <0.001 |
| Diabetes | 1.06 | 0.90-1.25 | 0.502 | 1.37 | 1.30-1.45 | <0.001 |
| Antihypertensive medication | 1.05 | 0.91-1.22 | 0.465 | 1.06 | 1.01-1.11 | 0.031 |
| Smoking |  |  |  |  |  |  |
| Never (reference) | - | - | - | - | - | - |
| Previous | 0.90 | 0.77-1.04 | 0.149 | 1.17 | 1.11-1.23 | <0.001 |
| Current | 0.94 | 0.77-1.14 | 0.531 | 1.71 | 1.60-1.82 | <0.001 |
| NIHSS on admission | 1.17 | 1.16-1.18 | <0.001 | 1.06 | 1.05-1.06 | <0.001 |

Patients who died within 30 days after onset were excluded from analysis of long-term survival. First 30 days were analyzed using logistic regression, while Cox regression was used for analyses of 30-day survivors.

OR: odds ratio, HR: hazards ratio, CI: confidence interval

## S9. Multivariable adjusted analyses of risk of short- and long-term mortality after stroke in women

|  | Short-term survival  n= 20 172 | | | Long-term survival  n= 18 811* | | |
| --- | --- | --- | --- | --- | --- | --- |
|  | OR | 95% CI | P value | HR | 95% CI | P value |
| Onset |  |  |  |  |  |  |
| Known-onset stroke (reference) | - | - | - | - | - | - |
| Wake-up stroke | 1.10 | 0.93-1.30 | 0.282 | 1.03 | 0.97-1.10 | 0.356 |
| Unknown-onset stroke | 0.90 | 0.74-1.11 | 0.338 | 1.18 | 1.11-1.27 | <0.001 |
| Age | 1.08 | 1.07-1.09 | <0.001 | 1.10 | 1.09-1.10 | <0.001 |
| Atrial fibrillation | 1.18 | 1.03-1.35 | 0.019 | 1.28 | 1.22-1.35 | <0.001 |
| Previous MI | 1.33 | 1.11-1.60 | 0.002 | 1.30 | 1.21-1.40 | <0.001 |
| Diabetes | 1.13 | 0.95-1.34 | 0.180 | 1.33 | 1.25-1.42 | <0.001 |
| Antihypertensive medication | 0.92 | 0.81-1.06 | 0.240 | 1.07 | 1.02-1.13 | 0.009 |
| Smoking |  |  |  |  |  |  |
| Never (reference) | - | - | - | - | - | - |
| Previous | 1.14 | 0.97-1.33 | 0.105 | 1.22 | 1.15-1.29 | <0.001 |
| Current | 1.32 | 1.08-1.60 | 0.005 | 1.61 | 1.50-1.73 | <0.001 |
| NIHSS on admission | 1.18 | 1.17-1.19 | <0.001 | 1.05 | 1.05-1.06 | <0.001 |

Patients who died within 30 days after onset were excluded from analysis of long-term survival. First 30 days were analyzed using logistic regression, while Cox regression was used for analyses of 30-day survivors.

OR: odds ratio, HR: hazards ratio, CI: confidence interval

## S10. Characteristics of patients with missing information on the variables smoking and NIHSS.

|  | All included patients  n=68 025 | Patients with missing information | |
| --- | --- | --- | --- |
|  |  | Smoking  n=10 815 | NIHSS  n=12 702 |
| Age | 73.8 (13.2) | 78.1 (12.8) | 74.2 (13.5) |
| Female | 44.9 | 52.1 | 46.2 |
| Living arrangement |  |  |  |
| Lives with someone | 56.8 | 49.0 | 51.5 |
| Lives alone | 39.9 | 42.6 | 43.0 |
| Institution | 3.2 | 8.5 | 5.5 |
| mRS prior to stroke |  |  |  |
| 0-1 | 76.5 | 66.2 | 71.1 |
| 2-5 | 23.5 | 33.8 | 28.9 |
| Smoking |  |  |  |
| No | 44.7 | - | 44.3 |
| Previous | 31.8 | - | 31.3 |
| Current | 23.6 | - | 24.4 |
| Diabetes | 18.8 | 19.8 | 21.1 |
| Atrial fibrillation | 24.7 | 32.9 | 44.3 |
| Prior myocardial infarction | 13.4 | 14.2 | 31.3 |
| Antihypertensive drugs | 54.5 | 56.4 | 24.4 |
| NIHSS on admission | 3 (1-7) | 6 (2-13) | - |
| Thrombolytic treatment | 20.4 | 25.0 | 26.6 |

Age is presented as mean (SD), NIHSS as median (IQR), all other results as percentages.

>5% missing values

SD; standard deviation, mRS; modified Rankin Scale, NIHSS; National Institute of Health Stroke Scale, IQR; interquartile range
